# Media Effects on Students during SARS Outbreak

**DOI:** 10.3201/eid1105.040512

**Published:** 2005-05

**Authors:** Sheri L. Bergeron, Ana L. Sanchez

**Affiliations:** * Brock University, St. Catharines, Ontario, Canada

**Keywords:** Canada, health communication, media, SARS, mass communication

## Abstract

A few months after the 2003 severe acute respiratory syndrome (SARS) outbreak, a sample of Canadian undergraduate university students completed a questionnaire that showed that, despite believing media coverage of the outbreak was excessive, they had little anxiety about acquiring SARS. Additionally, 69% of participants failed a SARS-specific knowledge section of the questionnaire.

The 2003 outbreak of severe acute respiratory syndrome (SARS) underlined the importance of fast and accurate risk communication to the public. Several studies have attempted to evaluate the media's performance during the outbreak (1,2), and the general consensus is that the media coverage was excessive, sometimes inaccurate, and sensationalist (1–3). Whether this excessive coverage had a beneficial or detrimental effect on the public remains unknown. A logical assumption would be that, in response, the public would not only have high anxiety of acquiring SARS, but also would be more informed about the cause, symptoms, and other aspects associated with the syndrome. The purpose of this study was to determine, in an undergraduate university student population, preferences and use of various types of mass communication media, anxiety levels of acquiring the infection, and general knowledge of SARS.

## The Study

Following ethics approval by the Brock University Research Ethics Board in October 2003, a pilot survey of 30 students was conducted to validate a questionnaire and determine the sample size for the study. Results showed that approximately 30% of participants may have had some anxiety of acquiring SARS during the outbreak. Therefore, in a population of 13,000 students, a sample of 310 was calculated to obtain a measurable anxiety level (Epi Info 6.04b, Centers for Disease Control and Prevention, Atlanta, GA, USA).

The questionnaire consisted of 25 questions in different formats (multiple choice, 7-point scale, open-ended, and follow-up streamed questions) and collected information on the following topics: demographics; access to and use of the Internet, radio, television, magazines, and newspapers for general use and as a source for SARS information; perceived amount of media coverage; anxiety of acquiring SARS; and SARS-specific knowledge.

The study was conducted from October 2003 to January 2004 in 2 consecutive phases; pen and paper questionnaires were used in the first and a Web-based version was used in the second. For phase 1, students were randomly approached between classes on 2 different days and invited to participate in the study. For phase 2, flyers were distributed inviting students to visit a Web site that contained all pertinent information and a link to an interactive questionnaire accessible only to Brock students. The Web site was available until the target sample size was reached. Phases were designed in such a way that students could only participate once.

Data were compiled by using Microsoft Excel 2000 (Microsoft Corp., Redmond, WA, USA) and transferred into SPSS Version 11.1 (SPSS Inc., Chicago, IL, USA). Statistical analyses included frequencies, proportions, and t tests/analyses of variance to evaluate differences among stratified media groups and results from the SARS knowledge section.

In total, 314 students enrolled in the study (186 in phase 1 and 128 in phase 2), but 14 incomplete questionnaires were excluded, resulting in a final sample of 300 students. Of the 300 participants, 219 (73%) were women, and 213 (71%) were majoring in nonhealth-related areas. The average age was 21.1 years (SD = 4.7), with 93% between 18 and 23 years of age.

Assessment of access and usage of 5 forms of mass media communication showed that 89% used the Internet, 88% television, 77% radio, 56% newspapers, and 28%, magazines. The daily use of television, radio, and Internet was categorized as previously reported (4), and participants were grouped into light (<2 h/day), medium (2–4 h/day) or heavy users (>4 h/day). Most students were light users of all 3 media types; however, Internet users were more likely to be heavy users than the other 2 groups combined (odds ratio = 3.55, 95% confidence interval 1.8–7.0, p = 0.0000357).

The perceived amount of media coverage was measured on a 7-point scale. Stratified results showed that most students (92.5%) considered the media coverage excessive.

Levels of anxiety of acquiring SARS were measured on a 7-point scale; the average anxiety level was 3.2, with a median of 3. Furthermore, when results were aggregated into low anxiety (score ≤3) and high anxiety (score ≥4) groups, 57% of students reported low anxiety, and 43% reported high anxiety. Anxiety levels were similar between health and nonhealth majors and were not associated with use intensity of any type of media.

**Figure 1:**
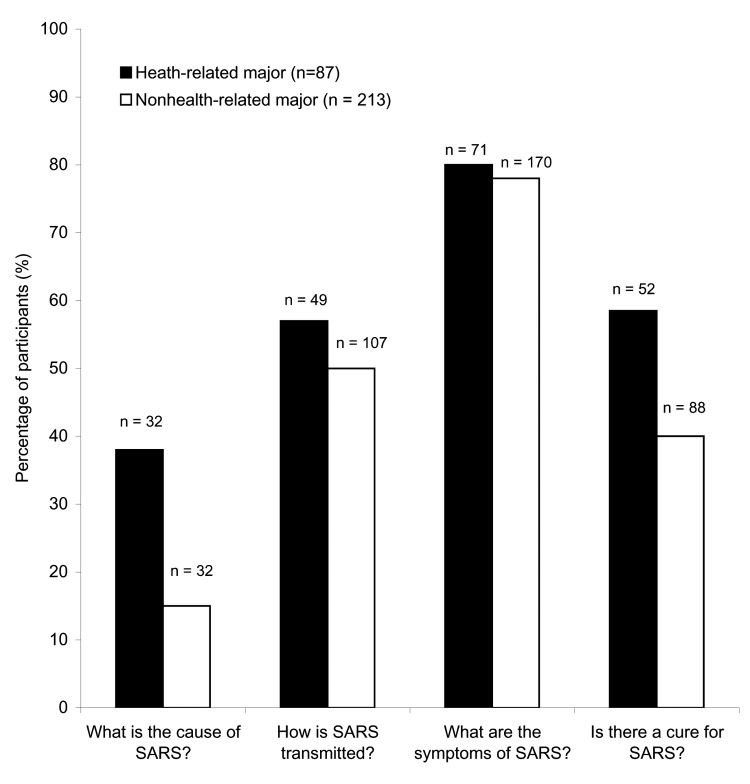
Percentage of participants who correctly answered each of the knowledge questions about severe acute respiratory syndrome (SARS) according to major (n = 300). *Statistically significant differences between health and nonhealth majors among the questions pertaining to the cause and treatment of SARS (p = 0.000 and p = 0.004, respectively).

Of the 300 participants, 206 (69%) failed the SARS-specific questions section (average 1.97, SD = 1.1); for which passing was defined as ≥3 correct answers of 4 questions, 1 each on cause, transmission, symptoms, and treatment of SARS. Health majors had a higher average score than nonhealth majors (t [degrees of freedom, df = 152] = -3.5, p = 0.001), particularly in regard to questions about the cause and treatment of SARS (Figure 1).

The average SARS-specific knowledge score was not statistically associated with access to and use of any type of media. However, a passing score was more common for those who used the Internet to obtain SARS-related information (t[df = 149] = 1.7, p = 0.088).

## Conclusions

Mass communication media are valuable resources for efficiently communicating risk information to the public. However, extensive collaboration among public health departments and media outlets is essential to deliver health information to all sectors of society (5,6). During the SARS outbreak, a deficient communication strategy among national and international public health agencies led to conflicting messages that created confusion and uncertainty in both the media and the general public (6). In reporting the events as they unfolded, the media communicated this confusion to the public. This study was undertaken only 5 to 8 months after the last known viral transmission (7) and sampled a particular sector of the population, young students attending a university in southern Ontario, 105 km from Toronto, the epicenter of the outbreak in Canada.

Most participants reported access to and use of several forms of media; the Internet as the most used, followed by television and radio. Newspaper and magazines were the least popular, which suggests that these forms of media are less appealing to young populations.

Overall knowledge about the cause, transmission, symptoms, and treatment associated with SARS was very low for this population. As expected, knowledge was higher among health majors but was not associated with any other variable. However, Internet use seemed to increase baseline SARS knowledge. A possible explanation for this observation is that, in contrast with television and radio, in which passive communication occurs, the Internet requires more participation, attention, and information processing as the user must search and choose to read the information. For a young population that prefers the Internet, this medium could be a great tool for delivering health messages.

When anxiety levels of acquiring SARS were assessed, the results did not support the assumption that the media created anxiety in this young population. The only predictors of high anxiety levels were sex (women) and area of residence in the greater Toronto area. Although anxiety levels for older age groups have not been studied, this finding may suggest that younger persons have different perceptions of health risks and that health messages should be designed with these differences in mind.

**Figure 2:**
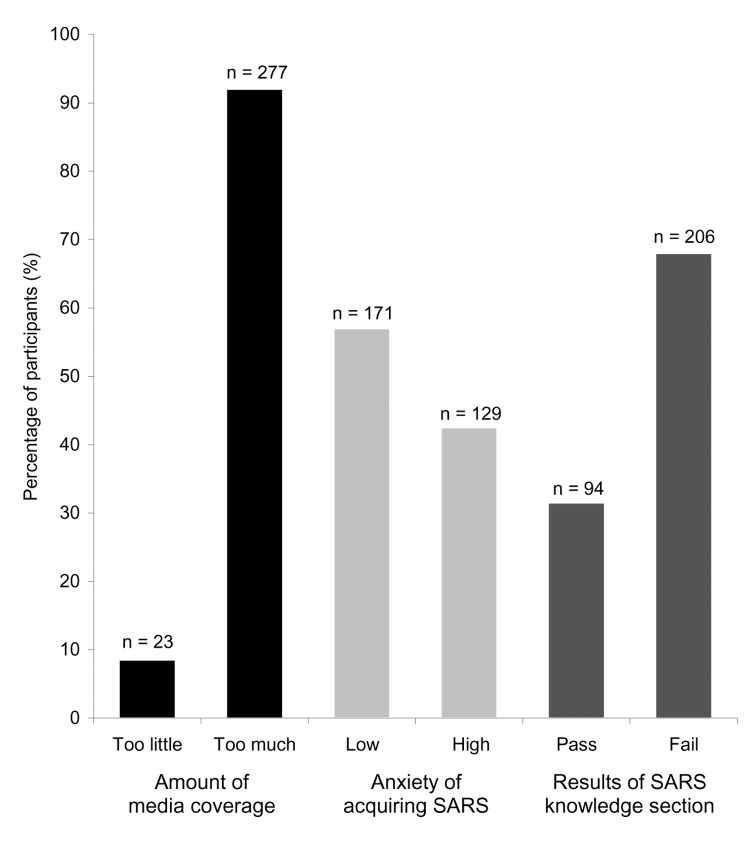
Summary findings of participants' perceptions about media coverage, level of anxiety of acquiring severe acute respiratory syndrome (SARS) and responses to the SARS knowledge section (n = 300).

In summary, this study showed that predetermined assumptions did not hold true for a young population. Despite believing that media coverage had been overdone, they reported low anxiety of acquiring SARS and showed poor knowledge of this emerging infectious disease (Figure 2). The discrepancy between the amount and type of information dispersed by the media and what was actually absorbed by the young population suggests that mere exposure to copious information is not enough to strengthen knowledge or elicit feelings that would induce persons to modify behavior.
